# Group-based positive psychotherapy for people living with acquired brain injury: a protocol for a feasibility study

**DOI:** 10.1186/s40814-024-01459-7

**Published:** 2024-02-21

**Authors:** Zoe Fisher, Susannah Field, Deb Fitzsimmons, Hayley Hutchings, Kym Carter, Daniel Tod, Fergus Gracey, Alec Knight, Andrew H. Kemp

**Affiliations:** 1 Community Brain Injury Service, Morriston Hospital, Swansea, UK; 2 Health and Wellbeing Academy, Swansea University, Swansea, UK; 3 Centre for Health Economics, Faculty of Medicine, Health and Life Science, Swansea University, Swansea, UK; 4 Swansea Trials Unit, Faculty of Medicine, Health and Life Science, Swansea University, Swansea, UK; 5 Department of Clinical Psychology and Psychological Therapies, Norwich Medical School, University of East Anglia, Norwich, UK; 6 King’s Undergraduate Medical Education in the Community (KUMEC), Faculty of Life Sciences & Medicine, Centre for Education, GKT School of Medical Education, King’s College London, London, UK; 7 School of Psychology, Faculty of Medicine, Health and Life Sciences, Swansea University, Swansea, UK

**Keywords:** Acquired brain injury, Chronic conditions, Randomised controlled trial, Wellbeing, Positive psychotherapy

## Abstract

**Background:**

Acquired brain injury (ABI) and other chronic conditions are placing unprecedented pressure on healthcare systems. In the UK, 1.3 million people live with the effects of brain injury, costing the UK economy approximately £15 billion per year. As a result, there is an urgent need to adapt existing healthcare delivery to meet increasing current and future demands. A focus on wellbeing may provide an innovative opportunity to reduce the pressure on healthcare services while also supporting patients to live more meaningful lives. The overarching aims of the study are as follows: (1) evaluate the feasibility of conducting a positive psychotherapy intervention for individuals with ABI and (2) ascertain under what conditions such an intervention would merit a fully powered randomised controlled trial (RCT) compared to a standard control group (TAU).

**Methods and analysis:**

A randomised, two-arm feasibility trial involving allocation of patients to either a treatment group (positive psychotherapy) or control group (treatment as usual) group, according to a 1:1 ratio. A total of 60 participants at three sites will be recruited including 20 participants at each site. Assessments will be conducted at baseline, on completion of the 8-week intervention and 3 months following completion. These will include a range of questionnaire-based measures, psychophysiology and qualitative outcomes focusing on feasibility outcomes and participant experience. This study has been approved by the Wales Research Ethics Committee (IRAS project ID: 271,251, REC reference: 19/WA/0336).

**Discussion:**

This study will be the first to examine the feasibility of an innovative, holistic positive psychotherapy intervention for people living with ABI, focused on individual, collective and planetary wellbeing, and will enable us to determine whether to proceed to a full randomised controlled trial.

**Trial registration:**

ISRCTN12690685, registered 11th November 2020.

**Supplementary Information:**

The online version contains supplementary material available at 10.1186/s40814-024-01459-7.

## Background

Acquired brain injury (ABI) refers to any type of brain damage that occurs after birth, including damage caused by infection, disease, lack of oxygen or an external force to the head. ABI may be sub-divided into traumatic brain injury (TBI), caused by a physical force to the head, resulting in damage to brain tissue, or non-traumatic brain injury (non-TBI), which includes causes such as stroke, brain tumour, hypoxia and meningitis. ABI leads to a wide range of physical, psychological and behavioural impairments including, for example disability, fatigue, cognitive dysfunction, emotion dysregulation and behavioural impulsivity. Such impairment significantly impacts on psychological wellbeing and poses a barrier to rehabilitation [1]. In the UK, 1.3 million people live with the effects of brain injury, costing the UK economy approximately £15 billion per year, a figure that is equivalent to 10% of the annual NHS budget [2]. Underpinning dominant western healthcare models is the insidious narrative that a person’s condition may be cured, yet ABI is a chronic condition that requires holistic long-term management [3]. With regard to chronic conditions such as ABI, there is tendency for models of healthcare to be overly focused on reducing deficits and psychological distress. This is despite compelling evidence that the absence of distress and ill-health is not synonymous with wellbeing and evidence from population-based studies that good psychological wellbeing reduces the risk of morbidity and mortality (see [4] for review), and that it remains possible to experience wellbeing despite suffering [5]. For instance, a seminal review paper focusing on happiness and the neurological disorders [6] noted that interventions to improve happiness can lead to improvements in patient status relating to a variety of diseases including epilepsy, Huntington’s disease, multiple sclerosis, Parkinson’s disease, and stroke. Consistent with these early insights, Evans and colleagues ( [7–9]) found positive psychology to be a useful approach for improving wellbeing for people living with ABI. Promising findings have been published with interventions focusing on use of signature strengths, reflection on positive events, volunteering, and goal setting [10–13]. Control comparison conditions in these studies included treatment as usual (TAU) [10, 11, 13] or waitlist-control conditions [12]. Treatment as usual included a variety of treatments including individual psychotherapy and group work, cognitive behavioural therapy, motivational interviewing, setting goals for rehabilitation, psychoeducation focused on brain injury, social skills and meal planning and pharmacological psychiatric treatments for mood disorders. The content of usual care is not typically standardised and depends on services available and participant needs.

Our study differs from past research and adds value to the literature in several novel ways:

1. We have developed an innovative positive psychotherapy intervention for ABI [14, 15] that seeks to promote wellbeing in service users in a more comprehensive way, focused on the promotion of individual, collective, and planetary wellbeing and based on our own theoretical model of wellbeing [16–18]. Our intervention makes use of positive psychological techniques, but is broader in scope, drawing on the wider evidence base on how to promote wellbeing.
2. Our approach to delivering the intervention involves training service user mentors to co-deliver the intervention, providing a meaningful role for the mentors themselves as well as hope and inspiration for those allocated to the intervention.
3. Our study adopts a mixed-method approach encompassing a range of measures including quantitative and qualitative measures, psychophysiological measures of wellbeing, and a health economic component, providing a more holistic perspective and the foundation on which deeper insights may be realised.

### Aim

The overarching aims of the study are as follows: (1) evaluate the feasibility of conducting a positive psychotherapy intervention for individuals with ABI and (2) ascertain under what conditions such an intervention would merit a fully powered randomised controlled trial (RCT) compared to a standard control group (TAU).

### Objectives

Our primary objective is to assess the feasibility of the research using the standardised ACCEPT checklist [19], which encompasses areas like recruitment rate, compliance with the intervention, randomisation process, data collection and analysis procedures and research governance and trial management. We will also delve into participants’ experiences within the trial, focusing on the acceptability of procedures and their engagement with the intervention while also collecting feedback for potential refinements. Additionally, by analysing our comprehensive dataset that includes quantitative, qualitative, psychophysiological and health economic data, we seek to identify early indications of the intervention’s impacts.

## Methods

This protocol has been developed in line with the Standard Protocol Items: Recommendations for Interventional Trials (SPIRIT) guidelines for streamlining the development and reporting of trial protocols [20] and the extension of the Consolidated Standards of Reporting Trials (CONSORT) for randomised pilot and feasibility trials [21]. Participant recruitment and data collection began in October 2022, and the last patient visit is expected by the end of October 2023. An independent Trial Steering Committee and Data Monitoring Committee with public patient involvement have been formed to oversee trial monitoring and management.

### Participants

Participants (*N* = 60) and mentors (*N* = 6) with a confirmed diagnosis of ABI will be recruited across three participating local health board sites: Swansea Bay University Health Board (SBUHB), Hywel Dda University Health Board (HDUHB) and Cardiff and Vale University Health Board (CVUHB). Sample size was determined based on our clinical experience running similar interventions, which typically involve 10 participants and 2 mentors per group and 2 courses per year. Quantitative analysis on collected data will provide us with more guidance on which sample size calculation for a full-scale RCT will be based.

#### Inclusion criteria

- Confirmed diagnosis of ABI
- Ability to actively engage in the intervention as determined by their neuropsychological assessment scores and their treating clinician
- Living in the community
- Age 18 years or older
- Living within the catchment area of one of the participating health boards
- At least 3 months post injury at the point of recruitment, allowing time for spontaneous recovery and for the person to develop an awareness of their difficulties and the implications of this on their lives

#### Exclusion criteria

- Receptive or expressive language difficulties or extremely low memory function that may preclude people from engaging meaningfully
- Medical or psychosocial reasons (based on risk assessment by the referring clinician)
- Potentially disruptive to other group members, as determined by their treating clinician
- Not able to provide informed consent

#### Additional inclusion criteria for mentors

Mentors will be subject to the same inclusion criteria as participants. They will also be subject to the following additional inclusion criteria:

- Known to and recommended by their referring clinical team
- Demonstrated ability to be responsive and sensitive to the needs of others
- Good interpersonal skills
- Willing and able to commit to training as well as attending all eight treatment sessions

A total of six mentors will be recruited including two mentors per health board.

### Ethics

Full NHS ethical approval was received from the Wales Research Ethics Committee on 6th January 2020 [IRAS project ID: 271,251, REC reference: 19/WA/0336]. The study uses a risk adaptive approach for monitoring and oversight. The trial will be subject to medium intensity monitoring, comprising a self-review (electronic remote data review) of the investigator site file following recruitment at each site, a review of completed data captured on the case report forms and an annual monitoring visit by the trial manager with 10–20% source data verification.

### Design

The study will randomly allocate eligible patients to either the treatment (positive psychotherapy [PP] intervention) or control (TAU) at a 1:1 ratio with stratification by site and antidepressant use (yes/no) (see further details below under ‘Randomisation’). The choice of a TAU control condition was based on ethical considerations and also enabled comparisons with past research [10, 11, 13]. Data collection will take place at each of the three healthcare sites, capturing a diverse representation of patients and enhancing the generalizability of the findings beyond a single site.

### Recruitment procedures

A site principal investigator (PI) will be identified at each site prior to starting the trial. The site PI and clinical staff will act as referrers for the trial to facilitate the identification of all potential patients. Full lists of active patients will be reviewed against the inclusion and exclusion criteria. Discussions about the study will be initiated by a treating clinician who is known by the patient. Potentially interested patient participants will be provided with a detailed participant information sheet that includes full details of the research activities and time commitments. Patients will then be called for a one-to-one telephone conversation with the PI, trial coordinator or research assistant for a more in-depth explanation of the study, to answer any questions and (if relevant) to book in their consent appointment. Consenting participants and mentors will be screened by a member of the research team to ensure that they are suitable for inclusion. This will involve the following:

1. Cross-referencing against the eligibility criteria
2. Brief standardised cognitive assessments including the Repeatable Battery for Assessment of Neuropsychological Status (RBANS) and the St Andrews-Swansea Neurobehavioral Outcome Scale (SASNOS)

Any participants deemed ineligible will be contacted by the PI and given an explanation.

### Randomisation

Participants will be randomly allocated to the intervention or TAU using REDCap [22, 23]. An authorised person will access REDCap to determine allocated treatment once eligibility has been confirmed. The participant will be notified of the allocation and the days on which the intervention will be delivered (if randomised to the intervention arm). The randomisation algorithm will be designed to promote balance in the sample sizes of the intervention and control groups, although exact numbers may vary slightly due to stratification for site (i.e. 3 sites; aiming for 10 participants per site, per arm) and antidepressant use (i.e. yes/no; aiming for an equivalent number of participants prescribed antidepressants per site, per arm).

### Treatment as usual

Treatment as usual will involve assessment and case management from different members of the multidisciplinary team. Following this, person-centred treatment goals will be set to guide neurorehabilitation efforts, and depending on an individual’s needs, a variety of treatments may be offered, either individually or in group settings. These may include the following: (a) Strategies to compensate for or ameliorate cognitive, physical or communication challenges; (b) psychological therapies, such as cognitive behavioural therapy, acceptance and commitment therapy and mindfulness; (c) vocational rehabilitation and engagement in meaningful activities; or (d) groups designed to support reintegration into local communities.

### The intervention

Over the last few years, we have developed an 8-week-positive psychotherapy intervention [14, 15, 24] involving one session per week over the 8-week period. Our treatment manual has been reiterated several times based on our previous clinical experience of running this group, user feedback and developments in wellbeing science. The present study will aid in further refining our intervention and materials including a clinician manual and participant workbook. Table 1 provides a summary of session-by-session content across the 8-week intervention.

**Table 1:** Session-by-session summary of 8-week-positive psychotherapy course for people living with acquired brain injury^a^

Session no. and name	Summary of session content
Session 1: Living with difficult emotions	• Difficult emotions are understandable following brain injury • A series of exercises encourage participants to accept such emotions • Participants learn coping skills, e.g., a ‘defusion exercise’ • Also includes focus on mindful breathing exercises and self-compassion • Difficult emotions motivate reflection and/or need for helpful changes • ‘Snakes and ladders’ adaptation helps to make content memorable
Session 2: Identifying and using character strengths	• Discussion of the nature of character strengths • Participants discuss results from a character strengths questionnaire • Discussion of how participants might use their strengths • Positive impacts of character strengths use are emphasised • Positive self-statements are created on which a positive introduction is built • ‘Snakes and ladders’ framework used to reinforce relevant concepts
Session 3: Building positive emotion	• Positive emotions have psychological and health benefits • They also help to cope with stress and adversity • Participants complete a ‘Getting to Know Your Lemon’ mindfulness exercise • Discussion of psychological flow, learned optimism and existential gratitude • Activities include mindful eating exercise and the ‘three good things’ activity • Participants complete meaning and values clarification exercise • Snakes and ladders’ framework is again used to reinforce relevant concepts
Session 4: Connection between body and mind	• Participants learn about the mechanisms of the mind–body connection • Focus on positive health behaviours, e.g., healthy diet, sleep, exercise • Regulatory role of the vagus nerve, indexed by heart rate variability (HRV) • Other techniques, e.g., singing, meditation, cold showers improve HRV • Participants explore acute impacts of different activities on own HRV • Highlights need for self-care, contextualised by healthy mind–body connection
Session 5: Connection to others and the natural environment	• Focuses on connecting to others and nature, and links to health and wellbeing • Science behind these connections, and how to improve wellbeing • Responding to good news and events, e.g., active-constructive responding • Variety of gratitude exercises, loving kindness meditation, volunteering • Nature-based activities good for wellbeing (e.g., gardening) • Also good for the environment (e.g., pro-environmental behaviours) • Participants complete a ‘photo-journalist exercise’
Session 6: Meaning and purpose	• Meaning and purpose give us a sense of direction and motivation in life • May be enhanced and facilitated by focus on the self, collective and planet • Discussion of photos representing areas of meaning for participants • Areas of meaning linked to personal values as per exercise in Session 3 • Behaviour-intention gaps constrain the translation of values into action • Reflection on how those gaps might be overcome
Session 7: Translating values into action	• Recap on participant's strengths, values and important areas of meaning • Explore extent to which participants are living a values-based life • Participants identify and share areas where they are acting out their values • Areas where they could better connect with their values are explored • Participants set goals that support them to reconnect with some of their values • Thoughts and feelings serve as barriers / facilitators to acting out values • Return to the metaphor of ‘snakes and ladders’
Session 8: Behaviour change and managing the ups and the downs	• Focus on sustainable behaviour change • Participants refine goals identified in Session 7 • Challenges encountered when moving toward wellbeing are revisited • Recap on strategies to manage those challenges • Techniques and strategies that can be practised to support wellbeing reviewed • Game of ‘snakes and ladders’ played to reinforce challenges and opportunities • Variety of resources provided to help practice different techniques

^a^Table adapted from [14]

### Procedure

**Fig. 1:**
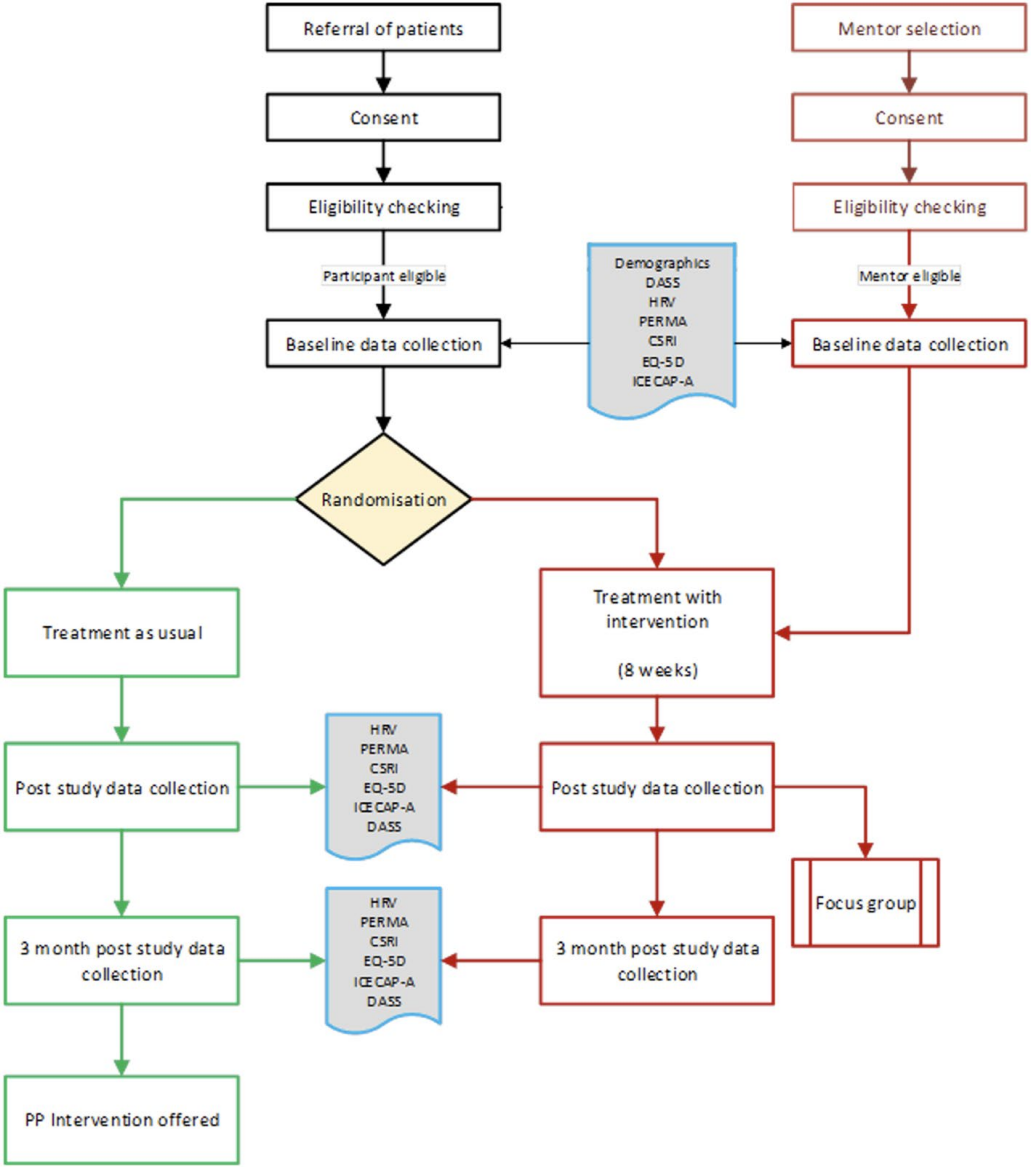
Flowchart of enrolment, interventions and assessments

The study will involve the following key stages (see also Fig. 1, Table 2):

1. *Referral*: Potential participants and mentors will be asked whether they would like to participate in the study and will be given the participant or mentor information sheet as appropriate.
2. *Consent*: Potential participants and mentors meet a member of the research team to discuss the study and provide consent.
3. *Eligibility*: Potential participants and mentors meet with the research assistant (under the supervision of a clinical psychologist) to determine eligibility for the study. If participants are deemed ineligible, they will be followed up by the PI and an explanation given.
4. *Baseline measures*: Eligible participants and mentors meet with the research assistant to complete baseline measures. A detailed description of the measures are provided below.
5. *Randomisation*: Participants will be randomly assigned to the TAU control group or the PP intervention group. Two mentors will be assigned to each of the three intervention groups based on availability and proximity.
6. *Treatment*: Participants and mentors attend the 8-week PP group or TAU control.
7. *Immediate follow-up*: All participants meet the research assistant to repeat quantitative measures over a 2-week period following the final session of the 8-week-positive psychotherapy intervention. Group attendees and mentors will also be invited to take part in participant and mentor focus groups, respectively, to gather data for qualitative analysis.
8. *Three-month follow-up*: All participants meet the research assistant to repeat quantitative measures a final time, 3 months following the final session for the intervention.

**Table 2:** Schedule of enrolment, interventions and assessments for participants (P) and their mentors (M)

Activity	Timepoints				
	**Eligibility checks**	**Baseline assessments**	**Psychotherapy sessions (1–8)**^**a**^	**Immediate follow-up**	**3-month follow-up**
Consent	P, M				
Eligibility checks	P, M				
• RBANS	P, M				
• SASNOS	P, M				
Data collection					
• HRV		P, M		P, M	P, M
• DASS-42		P, M		P, M	P, M
• PERMA Profiler		P, M		P, M	P, M
• EQ-5D-5L		P, M		P, M	P, M
• ICECAP-A		P, M		P, M	P, M
• Demographics		P, M			
• CSRI		P, M		P, M	P, M
Randomisation		P			
Intervention			P^a^, M		
Focus group				P^a^, M^b^	

*RBANS* refers to the Repeatable Battery for Assessment of Neuropsychological Status, *SASNOS* refers to St. Andrews-Swansea Neurobehavioral Outcome Scale, *HRV* refers to heart rate variability, *DASS-42* refers to the Depression, Anxiety and Stress Scales, PERMA-Profiler refers to the Positive emotion, Engagement, Relationships, Meaning, and Accomplishment Profiler, *EQ-5D-5L* refers to the EuroQual of life scale; *ICECAP-A* refers to the ICEpop CAPability measure for Adults, *CSRI* refers to an adapted version of the Client Service Receipt Inventory

^a^Intervention group only. TAU participants do not attend meetings or the focus group

^b^Intervention group participants and mentors attend separate focus groups

The wellbeing and care of participants will be prioritised throughout the study, providing support during data collection sessions, which involves the administration of measures of distress, well-being and quality of life. Any significant difficulties will be fed back to the wider clinical team to ensure that appropriate support is provided.

Any modifications to these procedures will be submitted to the Wales Research Ethics Committee for approval after which an updated version of the protocol will be made available at the ISRCTN Trial Registry (10.1186/ISRCTN12690685).

### Confidentiality

Patient records will be accessed by the local principal investigator (PI) and clinical teams in order to identify potential participants and gather necessary diagnostic information. Participants will be assigned a trial identifier to maintain anonymity, and personal identifiable information will be separated from outcome data in the REDCap database. Eligibility, safety and withdrawal data will be entered directly onto the REDCap database. The database will hold all meeting dates and attendance information. A secure Swansea Bay NHS server will contain patient identifiable information, linking individuals to their trial identifier. Access to identifiable information will be limited to authorized personnel, and all findings will be reported in a fully anonymised manner, with no personal details disclosed. Participants will be informed that direct quotes from focus groups may be used, but these will not identify individuals.

### Measures

Once participant eligibility has been confirmed, the following data will be collected from recruited participants.

### Cognitive assessment

Participants will complete paper-based standardised cognitive assessments: (a) the RBANS [25], a neuropsychological screening tool, commonly employed in ABI populations, which yield scores across five cognitive domains including immediate memory, visuospatial ability, language, attention and delayed memory, and (b) the St. Andrews-Swansea Neurobehavioral Outcome Scale (SASNOS) [26], a 49-item measure relating to a broad range of neurobehavioural difficulties people face when living with an ABI and measured on a 7-point scale ranging from ‘never’ to ‘always’. Response forms will be scored on paper, and index scores, confidence intervals and percentiles entered into dedicated case report forms (CRFs) on the REDCap database. The data will be summarised in a demographic table by trial arm to provide information to characterise the samples included in the study.

### Questionnaire-based measures

Participants will also complete a battery of questionnaires by verbally conveying their responses to a researcher who will type the participant’s response directly into the REDCap database. Paper questionnaires will be available for participants who wish to complete the questionnaires independently and as back-up in case of IT issues or participant processing difficulties. In these instances, the researcher will enter data onto the dedicated forms on the REDCap database following the assessment.

Questionnaires include the following: (a) the Depression, Anxiety and Stress Scales (DASS-42) [27], a 42-item measure of the severity/frequency of negative affective symptoms that are rated on a 4-point scale ranging from ‘never’ to ‘almost always’; (b) the EuroQual of life scale (EQ-5D-5L) [28], which measures five dimensions of health status including mobility, self-care, usual activities, pain/discomfort and anxiety/depression across five levels ranging from ‘no problems” to ‘unable/extreme problems’, alongside a visual analogue scale to provide a self-report of individual health status; (c) the ICECAP measure for adults (ICECAP-A) [29], which assesses five capabilities relevant to wellbeing including stability, attachment, autonomy, achievement and enjoyment; (d) the Positive emotion, Engagement, Relationships, Meaning, and Accomplishment (PERMA) profiler [30], a 23-item measure to assess flourishing across 5 domains (PERMA) as well as health, negative emotion, loneliness and overall happiness on a 11-point scale ranging from not at all/never to completely/always; and (e) an adapted version of the Client Service Receipt Inventory (CSRI) — Mental Health Version [31], which captures individual health service usage data.

### Psychophysiology

A Polar H10 heart rate sensor will be attached to a chest strap, placed around the chest wall and positioned below the pectoral muscles. The participant will then be placed in a seated position and left alone for 10 min, while heart rate variability data are collected. The Polar H10 device will be connected to the Elite HRV application [32], which will be installed on an iPad and connected to a secure NHS network, and the collected data will be exported as a plain text file to this network. The data file contains only millisecond timings between heartbeats and the unique study identification number. No personal information that can identify a participant will be included in this data file. Prior to data collection, participants will be asked some lifestyle questions regarding physical activity, time of last meal, alcohol intake, smoking status, sleep, height and weight. Prior work has demonstrated that the data collected from the Polar H10 devices are highly correlated with the hospital-grade electrocardiogram (*r* = 0.997) [33].

### Qualitative measures

Focus groups will be conducted with those participants allocated to the intervention group to facilitate a better understanding of study feasibility, clarify the components that participants like and dislike and help to determine what does and does not work. Questions regarding acceptability and participants’ experiences of the trial procedures and of the wellbeing intervention itself will be addressed through focus groups. A semi-structured interview schedule will cover topics including recruitment and data collection procedures, as well as experiences of participating in the group (see Supplementary information for a copy of this schedule). This will be implemented flexibly to facilitate group discussion and collection of rich and detailed data regarding participant experience, especially with regard to improvements needed for a future full-scale trial. The focus groups will be conducted by a female clinical trial coordinator who has a postgraduate background in psychology, facilitating a comfortable and confidential environment. Clinical staff members will be located nearby for governance purposes. Participants will be reminded about the purpose of the focus group, and that their data will be anonymized and potentially used for evaluation purposes. The interviewer will encourage group discussion by actively engaging participants in meaningful conversations and understanding the basis for differences in perspectives.

To ensure accuracy, the audio files of the focus groups will be transcribed using an orthographic approach. This transcription method includes incorporating verbal cues like ‘ah’, ‘um’ and other similar expressions. Additionally, grammatical correctness will be maintained to indicate pauses, the end of statements and exclamations. The focus groups will be transcribed verbatim, except for the exclusion of participant names, staff names and locations to safeguard anonymity.

### Analysis

As a feasibility trial, our primary endpoints are based on the criteria outlined in Table 3. We will also report descriptive statistics concerning adverse events, categorized by factors such as seriousness and severity, within each arm of the study. Adverse events will be continuously assessed as they are reported to determine their relevance to the trial design and the intervention at regular intervals.

**Table 3:** Criteria, measurement and the pass/fail system to determine study feasibility

Criteria	Measurement and justification	Fail	Pass
Recruitment across sites	Successful enrolment and recruitment rates will be monitored. Information will include number of participants recruited, number of participants declining and reasons for declining, and number of participants retained. Establishing a 50% recruitment rate threshold acknowledges the challenges inherent in recruiting individuals with ABI while also ensuring that the resources allocated to the study including time, funding and personnel are maximised. Recruitment rate will be determined as the % of target N recruited. The percentage of patients invited who agree to take part in the intervention will also be calculated	Issues at 1 + sites	All three sites recruit eligible patients
Recruitment rate (%)		< 50%	≥ 50%
Randomisation process	Randomisation process failures are defined as randomising an ineligible patient, a breakdown of the randomisation process and failure to adhere to the randomisation allocation. Maintaining a low threshold for randomisation process failures helps to ensure reliability and validity of trial outcomes	2 + issues with randomising	< 2 issues randomising participants
Intervention compliance (%) — clinicians	Adherence to the treatment manual as assessed by review of a content checklist, completed by the group facilitator at the end of every session at each of the three sites. Setting a threshold of 80% ensures a high standard of intervention fidelity across sites while still accounting for minor deviations that may occur due to contextual factors or facilitator styles	< 80%	≥ 80%
Intervention adherence (%) — participants	Attendance at a minimum of six sessions will be used to measure adherence. Full or partial completion of the two pieces of mandatory homework allocated to participants during session 1 and session 5 will also be documented. Setting the threshold at 75% ensures that participants are engaging in at least six of eight sessions of the intervention while also mindful of potential challenges participants may face such as scheduling conflicts and unexpected circumstances	< 75%	≥ 75%
Data collection	Success would be indicated if completion rates for post-intervention assessment and follow-up data collection reach a satisfactory threshold, taking into account practical considerations including participant compliance and capacity, versus representativeness and generalisability. Missing data will be reported	< 70%	≥ 70%
Attrition rates	Attrition will be calculated as the percentage of participants dropping out of the study relative to the numbers recruited. Reasons for withdrawal and loss to follow-up will be reported. Setting an attrition threshold at 40% acknowledges the potential challenges of participant retention in longitudinal studies	≥ 40%	< 40%

In the event that any of the feasibility criterion fail to meet the established targets for progression (Table 3), a comprehensive assessment will be undertaken that explores the reasons for the failure and determines the appropriate course of action. Based on that assessment as well as more general considerations relating to the detailed acceptance checklist [19], the research team will consider available options to address the feasibility challenges with the aim of optimising recruitment strategies and trial design. Ultimately, the decision to progress to a full-scale randomized controlled trial (RCT) will be based on a careful evaluation of the feasibility challenges in relation to the team’s capacity to run a future trial with confidence and achieve its objectives. The aim will be to strike a balance between addressing the identified challenges and ensuring the feasibility and success of the full-scale RCT. This approach will enhance the team’s ability to gather robust data, meet scientific standards and ultimately contribute valuable insights to the field of study.

These feasibility data will be complemented by inspection of additional quantitative, qualitative, psychophysiological and health economic data, as outlined in further detail below.

### Quantitative data

This feasibility trial will explore the most appropriate primary outcome for a fully powered RCT. Data analysis, following intention-to-treat principles, will focus on descriptive statistics and feasibility outcomes, consistent with the primary aims and objectives of the trial. Summaries, including completeness, for outcome measures and important demographic covariates will be reported by group. No missing data will be imputed. While clinical effectiveness will not be definitively assessed at this stage, we will explore hypotheses that measures of depression, anxiety and stress (as operationalised by the DASS) will diminish, and measures of PERMA and HRV will improve, using a split-plot ANOVA with group (intervention vs control) as a between-subjects factor across time (pre vs two follow-up assessments), a within-subject factor. All assessments will be summarised using two-sided tests and 95% confidence intervals where appropriate. Estimates of treatment effect size and intraclass correlations will be used to inform sample size considerations for a full-scale RCT.

### Qualitative data

The embedded qualitative aspect of the study will be based on a critical realist perspective, in which the acceptability and experience of the trial and intervention are embedded in the social contexts of participants. This means data analysis will attend to both the manifest content of interviews to ascertain concrete feedback regarding procedures, as well as being sensitive to the contextual features that shape people’s experiences and views.

We will adopt a pragmatic, reflexive and critical realist stance, analysing the qualitative data using reflexive thematic analysis (RTA) [34, 35]. Data analysis will explore key themes/codes using organic and open-ended coding, theme refinement and recursivity of analytic phases, facilitating prolonged and deep engagement to produce a meaningful and useful analysis. Coding will proceed according to the steps of RTA. We will seek sufficient coherence across codes to address the study aims regarding acceptability and experience of trial procedures and the intervention within the sample. However, we will not set a cut point for sampling or analysis based on the concept of data saturation, consistent with RTA guidelines [36]. Coding will be conducted by one researcher after which codes will be reviewed and discussed with other members of the team who will sense-check themes and offer alternative interpretations of the data with the aim of developing richer meanings.

In line with a critical realist approach, attention will be paid to how perspectives might be shaped by social context — for example whether differences emerge across study sites or social demographics. This approach will help to understand the different ways in which social factors might impact on procedures for the conduct of a future full trial. Prolonged and deep engagement with the data, transparency of the analytic process, support for reflexive practice through use of a journal and reflexive supervision, charting of the coding process and clear characterisation of participants and data collection contexts will contribute to achieving credibility, transferability, dependability and confirmability of data and methods, consistent with Lincoln and Guba’s evaluative criteria [37].

### Health economic evaluation

We will examine the feasibility of collecting the data required for a full economic evaluation to determine the cost-effectiveness of PP compared to TAU in a future RCT. We will provide a provisional description of the resource use and costs of the PP intervention compared to TAU from an NHS and personal social services (PSS) perspective. A cost consequence analysis will be undertaken, and the costs of PP will be tabulated and described against TAU, to inform the costs and outcomes that will be the most relevant in a future definitive trial.

### Analysis of mentor data

The primary aim of this study is to explore the feasibility aspects of the study, including the recruitment and acceptability of recruiting two mentors per site. We will also calculate effect sizes from the data obtained from the six mentors, and these calculations will be exploratory in nature, given the context of a feasibility study. The primary purpose here is to gather preliminary data that will inform the design of a full-scale randomized controlled trial. We will also conduct qualitative evaluations to delve into themes that emerge from focus groups conducted with the mentors. This qualitative aspect will provide deeper insights into the mentors’ experiences and perspectives, which quantitative data alone might not fully capture. The feasibility of recruiting and engaging two mentors per study is a key aspect we aim to assess. Understanding whether this approach is practical and acceptable to the mentors themselves is vital for the successful implementation of the full-scale trial.

### Data preservation and accessibility

All patient-identifiable information stored in the NHS (with the exception of entries in clinical notes) will be destroyed within 5 years of the start of the study. Fully anonymised data will be made open access once the trial ends, consistent with developments in the open science movement.

## Discussion

This feasibility study will provide a comprehensive evaluation of the essential components necessary for the successful execution of a future trial. These components include recruitment, compliance, randomization, data collection and analysis procedures, research governance and trial management. By conducting this study, valuable insights and knowledge will be generated, serving as the foundation for determining the feasibility of conducting a future definitive randomized controlled trial.

### Supplementary Information

**Additional file 1: Supplementary information.** Semi-structured interview schedule.

## Data Availability

Not applicable.
